# Supplementary material for: Metaplastic Barrett's oesophagus represents reversion to a developmental-like epithelial cell state
Source: Development. 2025 Nov 20;152(22):dev204735. doi: 10.1242/dev.204735 (PMC12669961; doi:10.1242/dev.204735)
Supplement: Supplementary information [file develop-152-204735-s1.pdf]

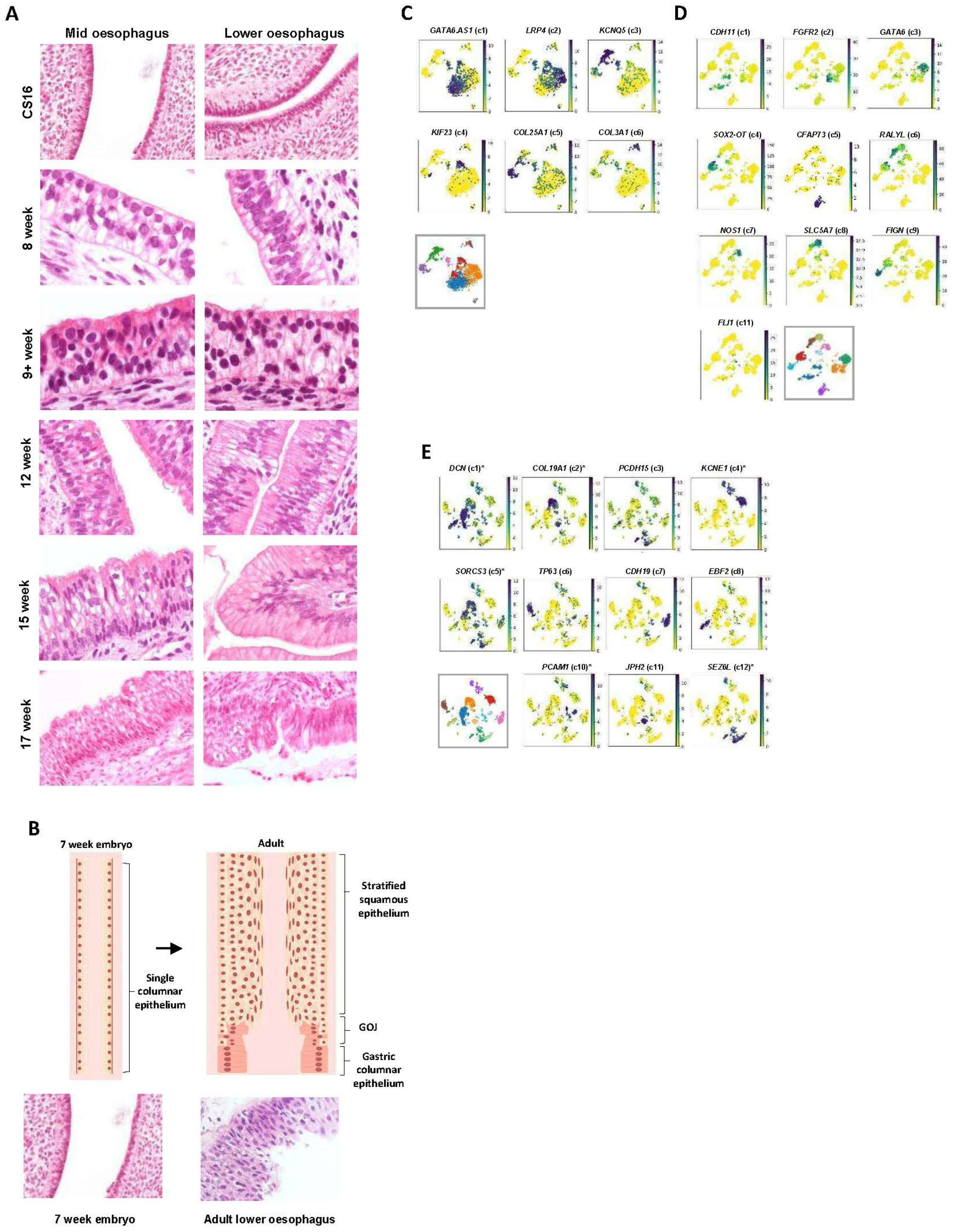

Fig. S1. Characterisation of the developing human oesophagus. (A) Examples of H&E stained embryonic/fetal tissues at the indicated developmental stages. The CS16, 12 week and 17 week H&E images in A (mid oesophagus) are from Fig. 1. The CS16 image is also shown in B (7 week embryo). (B) Schematic illustration of the changing nature of the oesophageal epithelial layer in the embryo and adult. Representative H&E staining of the epithelial layers is shown below each stage. (C-E) tSNE plots with the indicated cluster marker gene expression superimposed for week 7 (C), week 9 (D) and week 15 (E). Note that week 7 clusters 7 and 8, week 9 cluster 10 and week 15 cluster 9 lack distinct marker genes which highlight the cluster. Asterisks in E indicate genes that demarcate one cluster but are also found in additional clusters.

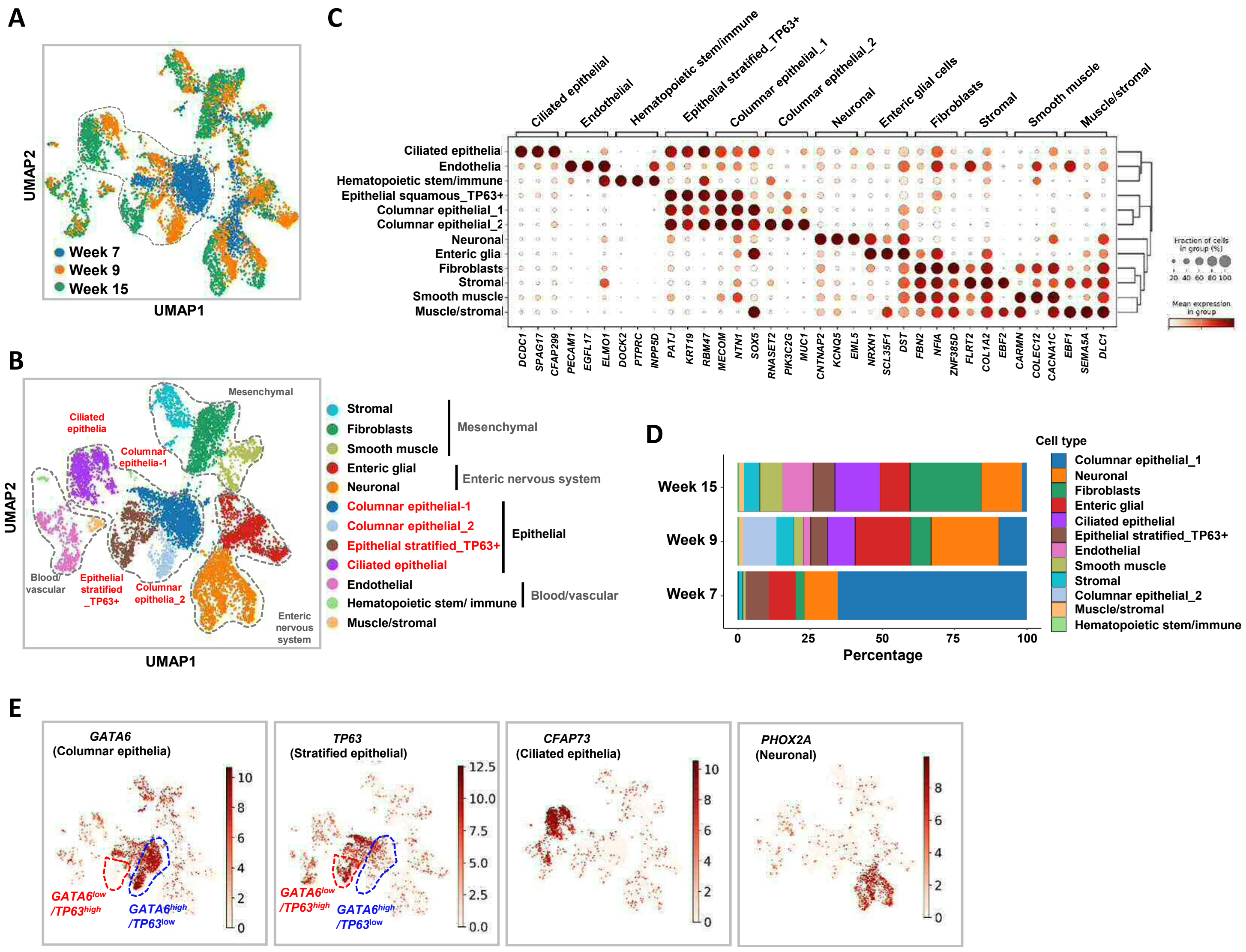

Fig. S2. Characterisation of the cell populations in the developing oesophagus. (A and B) UMAPs of all the cell populations from the combined week 7, 9 and 15 samples. The locations of cells originating from each time point (A) and the major cell types (B) are projected on top of the clusters and grouped according to similarity. (C) Dotplot of the relative expression of three representative markers for each of the cell clusters annotated in part B. The fraction of cells expressing each marker and relative average expression levels (column normalised) are represented by the size and intensity respectively, of each dot. (D) Percentage of each of the major cell types found at each developmental timepoint. (E) Relative expression (indicated by scale bars) of the indicated genes projected on cells in the UMAP in parts A and B.

Development • Supplementary information

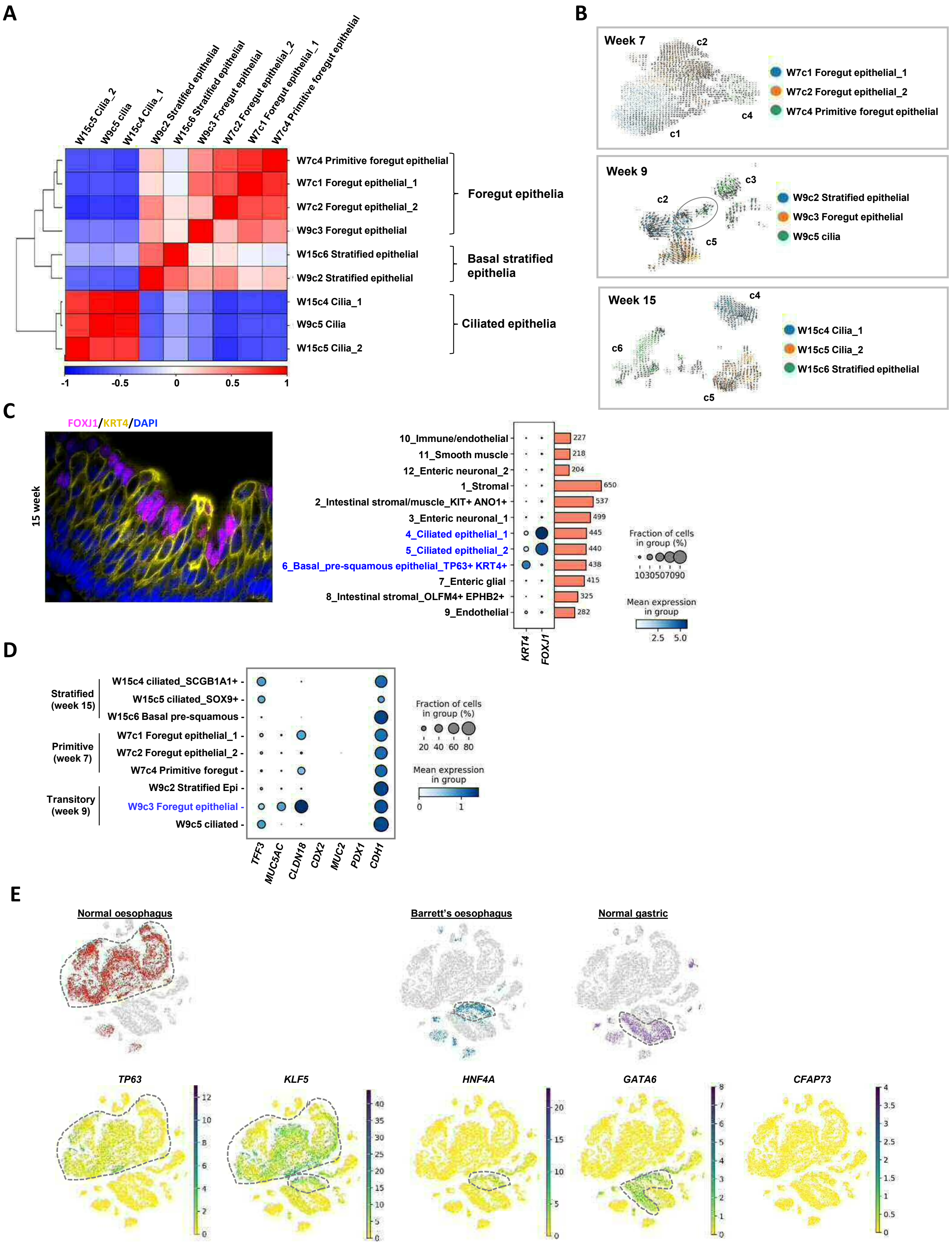

Fig. S3. Inter relationships between epithelial cell clusters. (A) Pearson's correlation plot comparing gene expression profiles of the epithelial cell clusters across developmental time points. Broad categories of epithelial cell types are indicated. (B) RNA velocity (scVelo) analysis of the epithelial cell clusters at each time point superimposed on tSNE plots (same plot as Fig. 2B but with more arrows). (C) Immunohistochemistry (left) and dotplots (right) showing the expression of *FOXJ1* and *KRT4* in the epithelial layer of 15 week embryos. (D) Dotplots showing the expression of the intestinal marker genes in the oesophageal epithelial cell clusters at different stages (gastric-*TFF3, MUC5AC, CLDN18*; early intestinal- *CDX2*; goblet cell- *MUC2*; pancreatic-*PDX1*; pan-epithelial *CDH1*). (E) UMAPs of snRNA-seq data from the cell populations found in the adult upper GI tract and BO (Nowicki-Osuch et al., 2021). Cells corresponding to the different tissue or disease types are shown (top) and the expression of the indicated genes are superimposed on top of the UMAP (bottom).

Development • Supplementary information

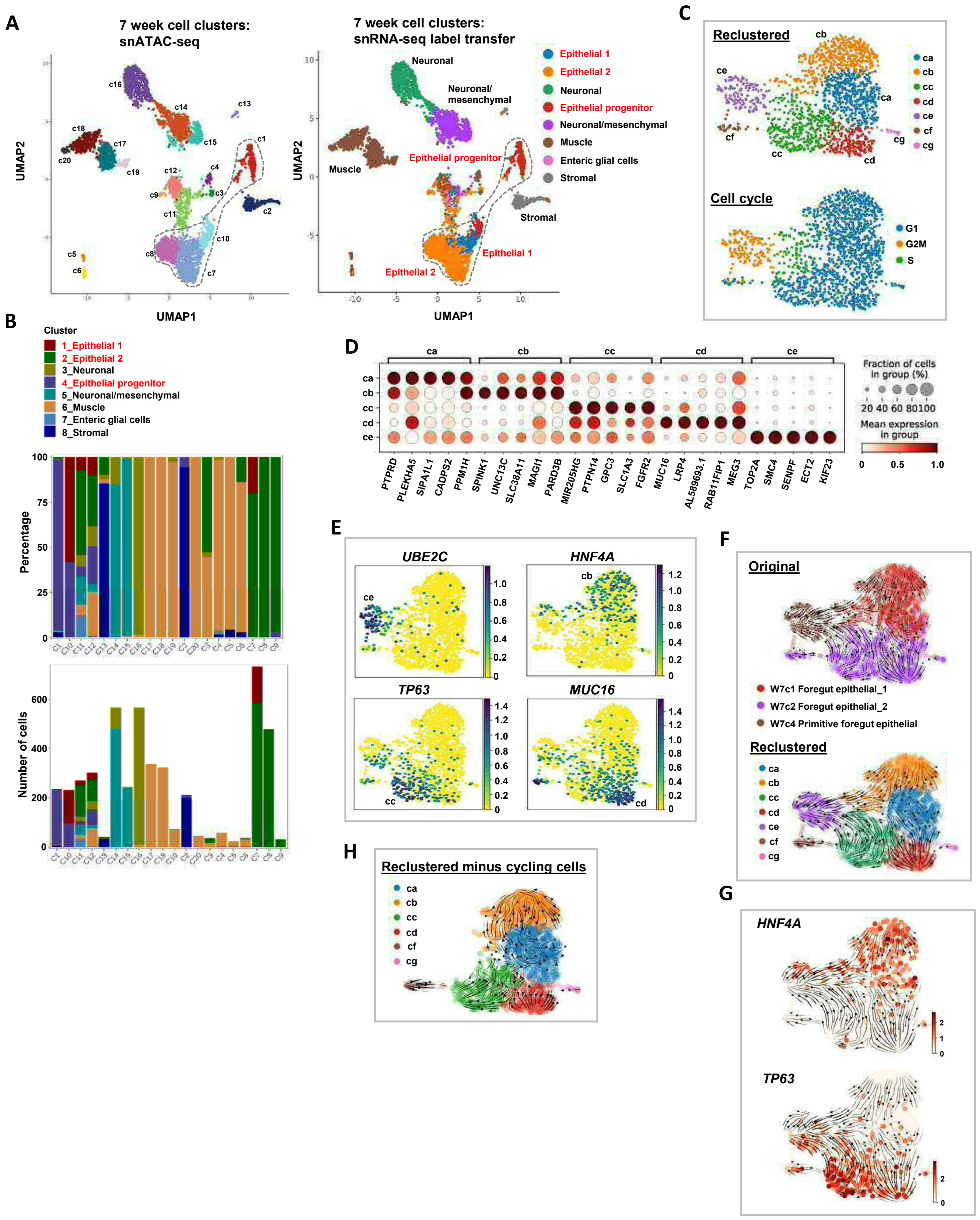

Fig. S4. Development regulatory networks of the primitive stage epithelial cell populations. (A) UMAPs derived from snATAC-seq data showing the cell populations in 7 week embryos. Cells are clustered according to snATAC-seq signal (top), annotated based on label transfer from snRNA-seq (right). Note that the enteric glial cells mapped to cluster c11 but only represented a small proportion of the entire cluster, and along with clusters c3 and c12, cluster c11 contained numerous cell types, potentially due to technical reasons. These clusters were located close to the small clusters c9 and c4 and these five clusters could not be unambiguously defined by label transfer. (B) Proportion of cells found in each of the snATAC-seq-derived 7 week clusters matching with each snRNA-seq derived 7 week cluster after gene expression integration (top). Total numbers of cells in each cluster are also shown (bottom). (C) UMAPs derived from snRNA-seq data in 7 week embryos following reclustering into 7 clusters (top). Cell cycle stage is superimposed on this UMAP (bottom). (D) Dot plot showing the five top marker genes for clusters ca-ce. (E) UMAPs of epithelial cells in 7 week embryos with the indicated gene expression levels superimposed. (F-H) RNA velocity analysis showing original clusters (see Fig. 2D) (F, top) and reclustered epithelial cells (F, bottom), *HNF4A* or *TP63* superimposed on the UMAP (G) or directionality in velocity following removal of cycling cells (H).

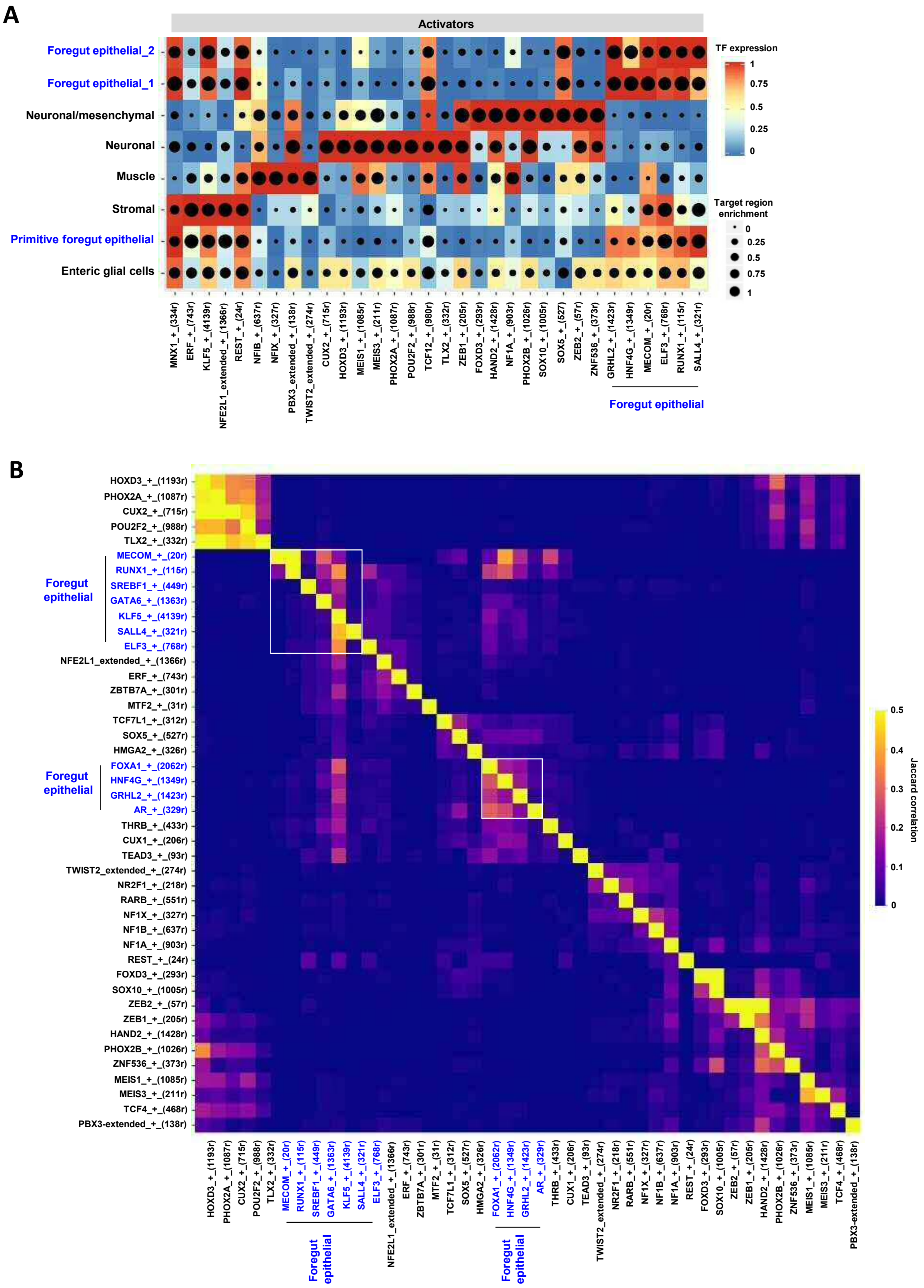

Fig. S5. Gene regulatory networks (GRN) in the primitive stage cell populations. (A) Top scoring GRNs in each of the 7 week cell clusters (eRegulons correlation coefficient above 0.70 or below -0.75). Transcription factors and number of linked target genes (in brackets) are shown with relative TF expression levels colour coded, and target region enrichment in each cluster is shown by the size of dots. Cell states are labelled according to label transfer from snRNA-seq.(B) Heatmap depicting an extended number of GRNs found in the 7 week cell clusters. Jaccard correlation coefficients between the different GRNs are depicted with foregut epithelial GRNs boxed.

Development • Supplementary information

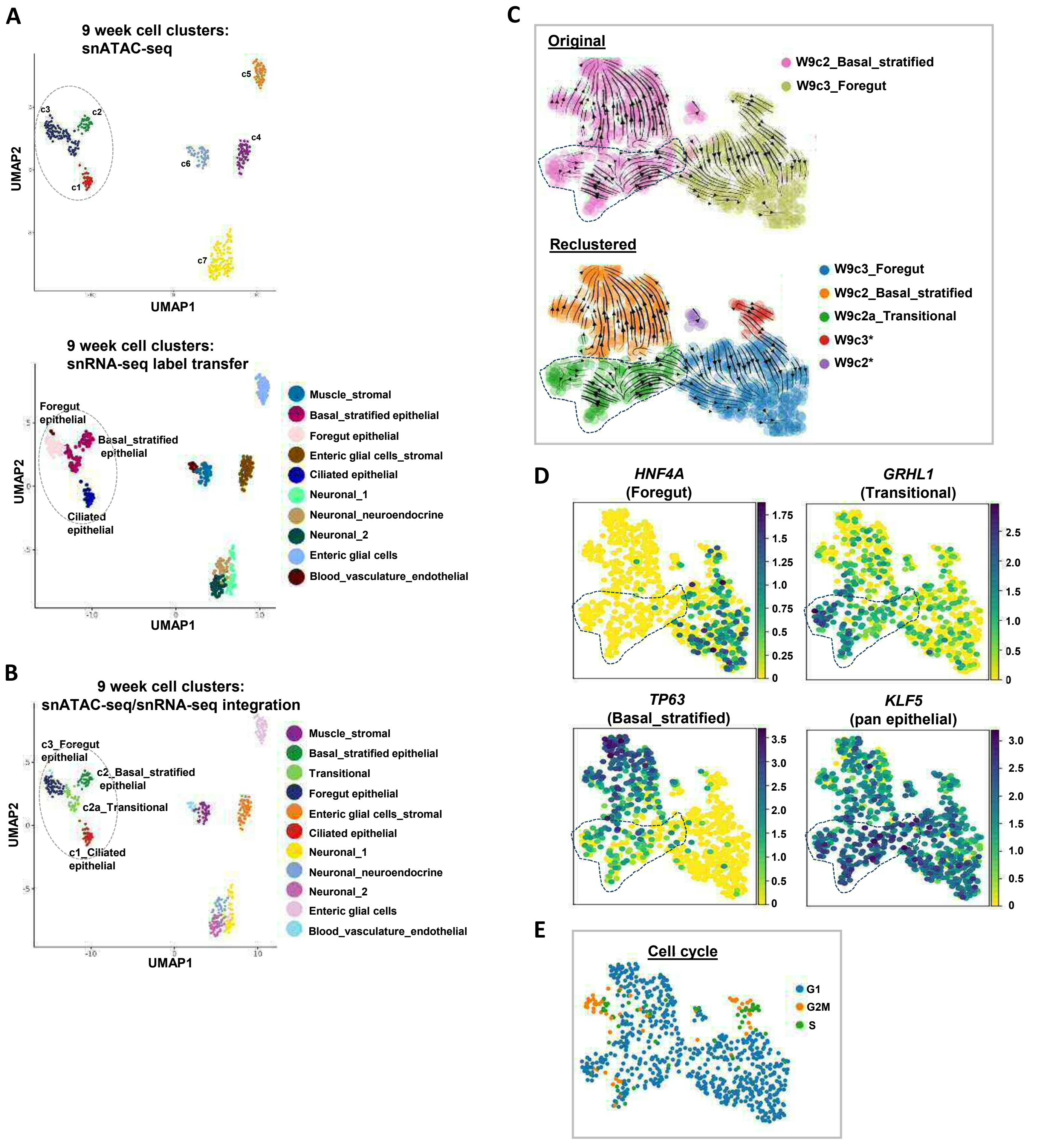

Fig. S6. Development regulatory networks in the columnar to squamous epithelial transition. (A) UMAPs derived from snATAC-seq data showing the cell populations in 9 week embryos. Cells are clustered according to snATAC-seq signal (left), annotated based on label transfer from snRNA-seq (right). (B) UMAPs derived from snATAC-seq data from the cell populations in 9 week embryos, reclustered after label transfer from snRNA-seq. The new transitional population c2a is added to the epithelial cell clusters. Epithelial cell clusters are enclosed within the by dotted ellipses. (C) RNA velocity analysis from snRNA-seq data showing original c2 and c3 epithelial cells clusters (top) and 5 new clusters when these cells are reclustered (bottom). The transitional cluster c2a is highlighted. (D and E) UMAPs of epithelial cells in clusters c2 and c3 in 9 week embryos (reclustered in C) with the indicated TF marker gene expression levels superimposed (D) or cell cycle phases indicated (E).

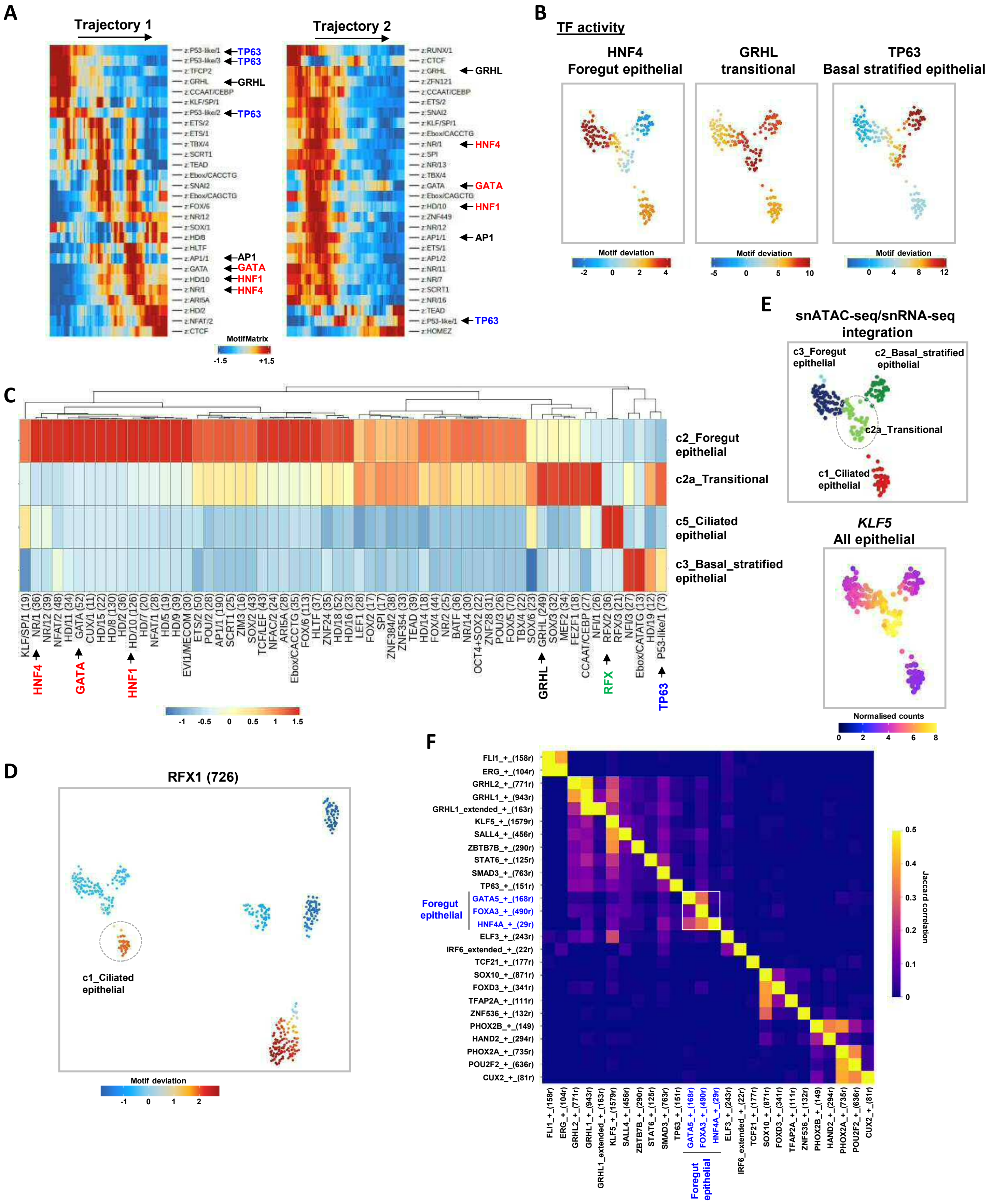

Fig. S7. Regulatory networks in week 9 epithelial cells. (A) Motif deviation score across pseudotime with max and min value limited to 1.5 and -1.5 respectively, showing all motif labels across the trajectories depicted in Fig. 4C and D.(B) Transcription factor binding motif deviation scores for HNF4, GRHL and TP63 in individual cells projected on the epithelial population snATAC-seq-derived UMAP. (C) Heatmap showing the relative enrichment of the indicated transcription factor binding motifs in each of the epithelial cell clusters (scale bar shows scaled hypergeometric enrichment of a peak annotation). Motifs discussed in the text are highlighted. (D) Transcription factor binding motif deviation scores for RFX transcription factors in individual cells projected on all of the cell clusters in the snATAC-seq-derived UMAP. (E) UMAP of the epithelial cell clusters derived from snATAC-seq (top) showing the expression (gene integration scores from snRNA-seq) of *KLF5* (bottom). (F) Heatmap depicting an extended number of GRNs found in the 9 week cell clusters. Jaccard correlation coefficients between the different GRNs are depicted with foregut epithelial GRNs boxed.

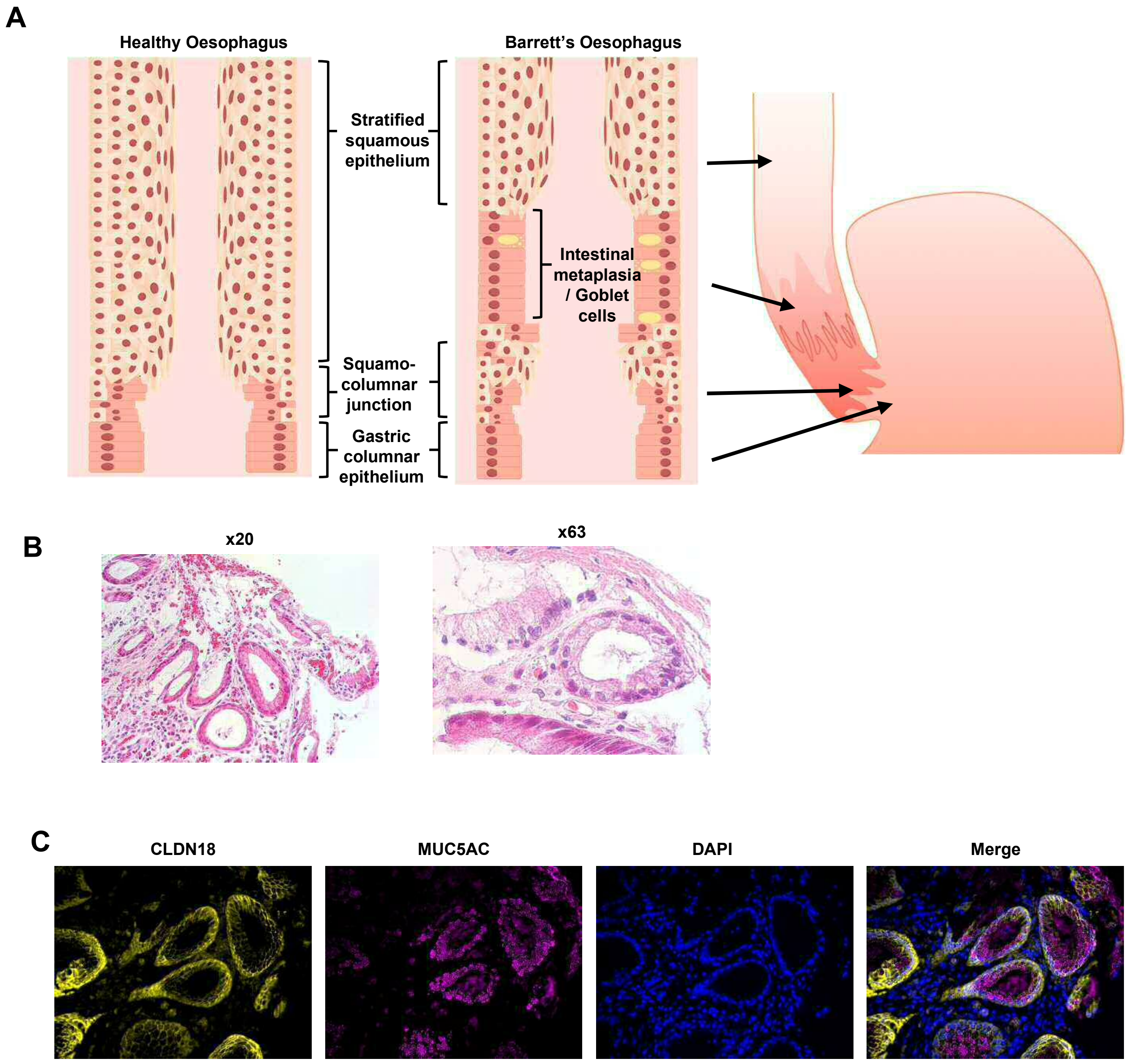

Fig. S8. Barrett's oesophagus tissue architecture characterisation. (A) Schematic views of the cellular organisation of adult oesophagus in healthy and Barrett's patients. (B) H&E staining of Barrett's sample used to generate snATAC-seq at 20x (left) and 63x (right) magnification. (C) ISH analysis of the Barrett's marker proteins CLDN18 and MUC5AC in Barrett's samples. Nuclei are shown by DAPI staining.

Development • Supplementary information

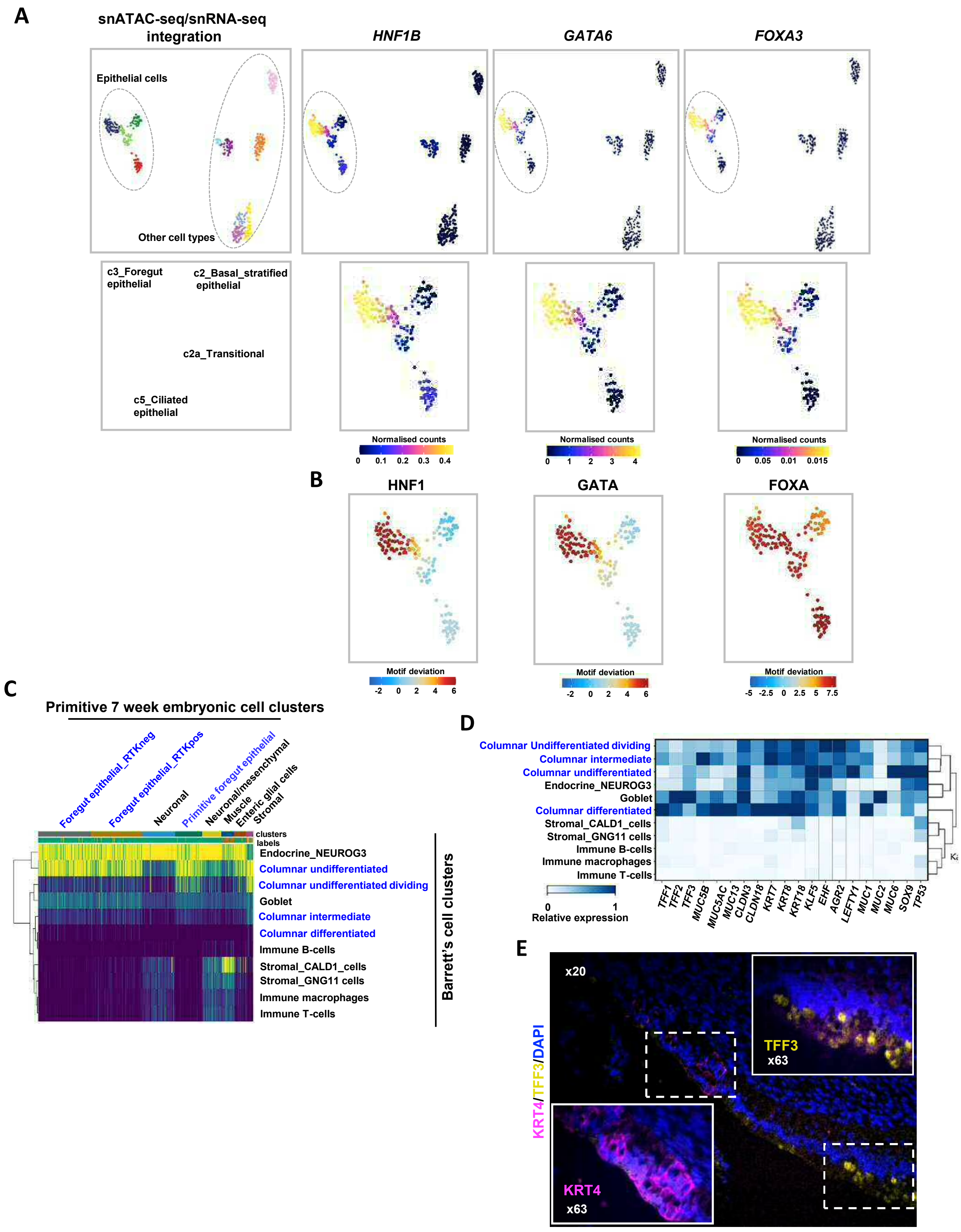

Fig. S9. Barrett's oesophagus resembles early developmental cell states. (A) UMAPs of week 9 developmental clusters derived from snATAC-seq (see Fig. 4) showing cluster identifications (leftmost panels) or with the expression of the indicated transcription factors (by gene integration from RNAseq) projected on top of these (right panels). Epithelial cell populations are highlighted below the complete heatmaps. (B) Transcription factor motif scores in each cell projected on top of the epithelial cell clusters. (C) Heatmap showing similarity scores between each cell in the 7 week developmental clusters (x-axis) and the corresponding cell types found in Barrett's cell clusters (y-axis). (D) Heatmap showing the relative expression of the indicated Barrett's marker genes (scaled for each column) in each of the Barrett's sample scRNA-seq derived clusters. E) Immunofluorescence staining of 9 week oesophageal tissue for the nuclear marker DAPI, the squamous epithelial marker KRT4 (pink) and also TFF3 (green) which highlights secretory columnar cells in the lower oesophagus. Higher magnification insets are shown of the upper (left) and lower oesophagus (right).

Development • Supplementary information

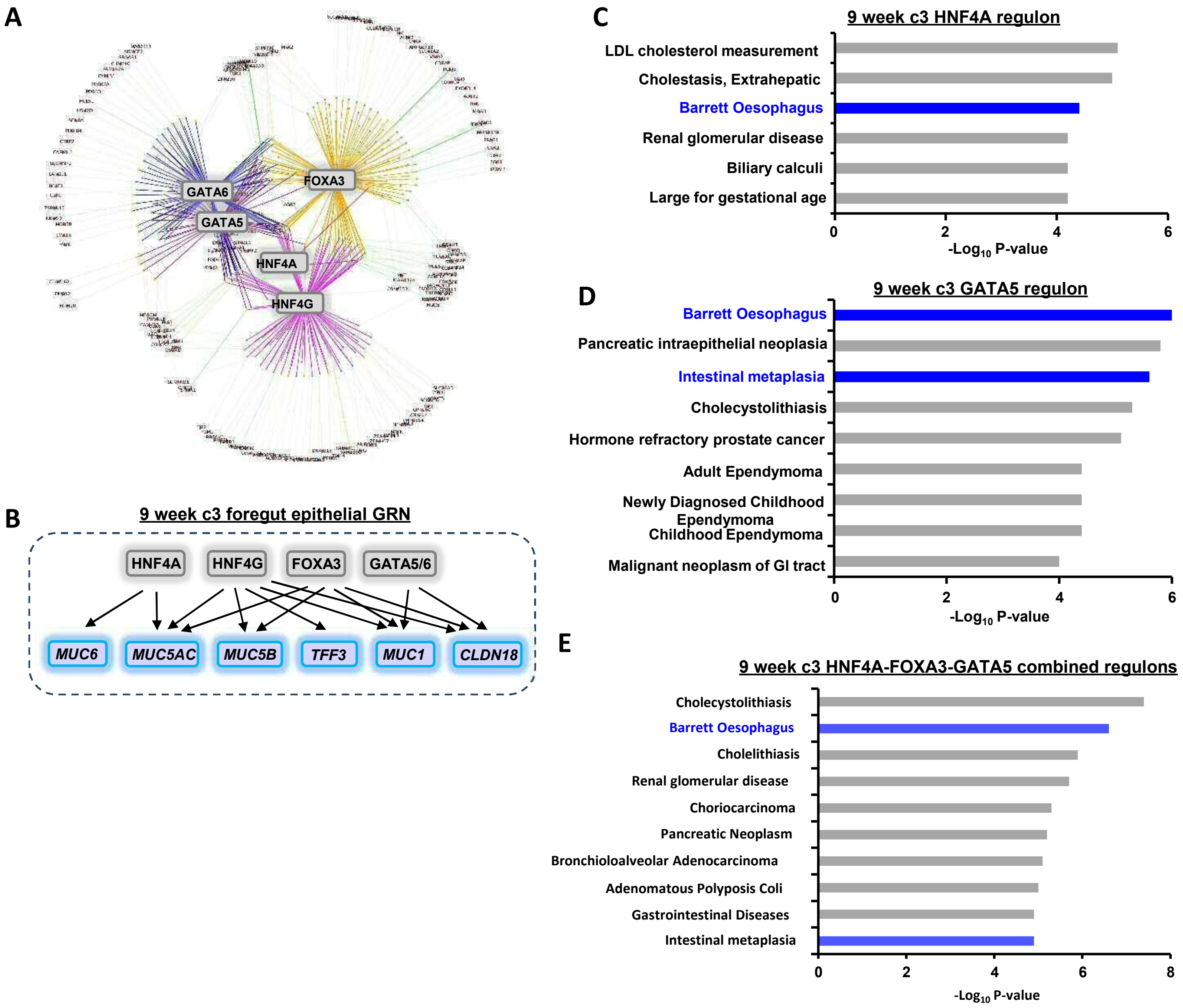

Fig. S10. Barrett's-like GRNs in the foregut epithelial cells of transitory state embryos. (A) Broad view of the gene regulatory networks controlled by the GATA-FOXA-HNF4 TF axis. (B) Regulatory links derived from the week 9 foregut epithelial cluster GRNs, depicting upstream TFs (top) and their links to Barrett's associated genes (bottom). (C-E) Enriched DisGeNET GO terms for the HNF4A regulon (29r)(C), GATA5 regulon (168r)(D) and combined HNF4A (29r), FOXA3 (490r) and GATA5 (168r) (E) in 9 week c3 foregut epithelial cells.

Development • Supplementary information

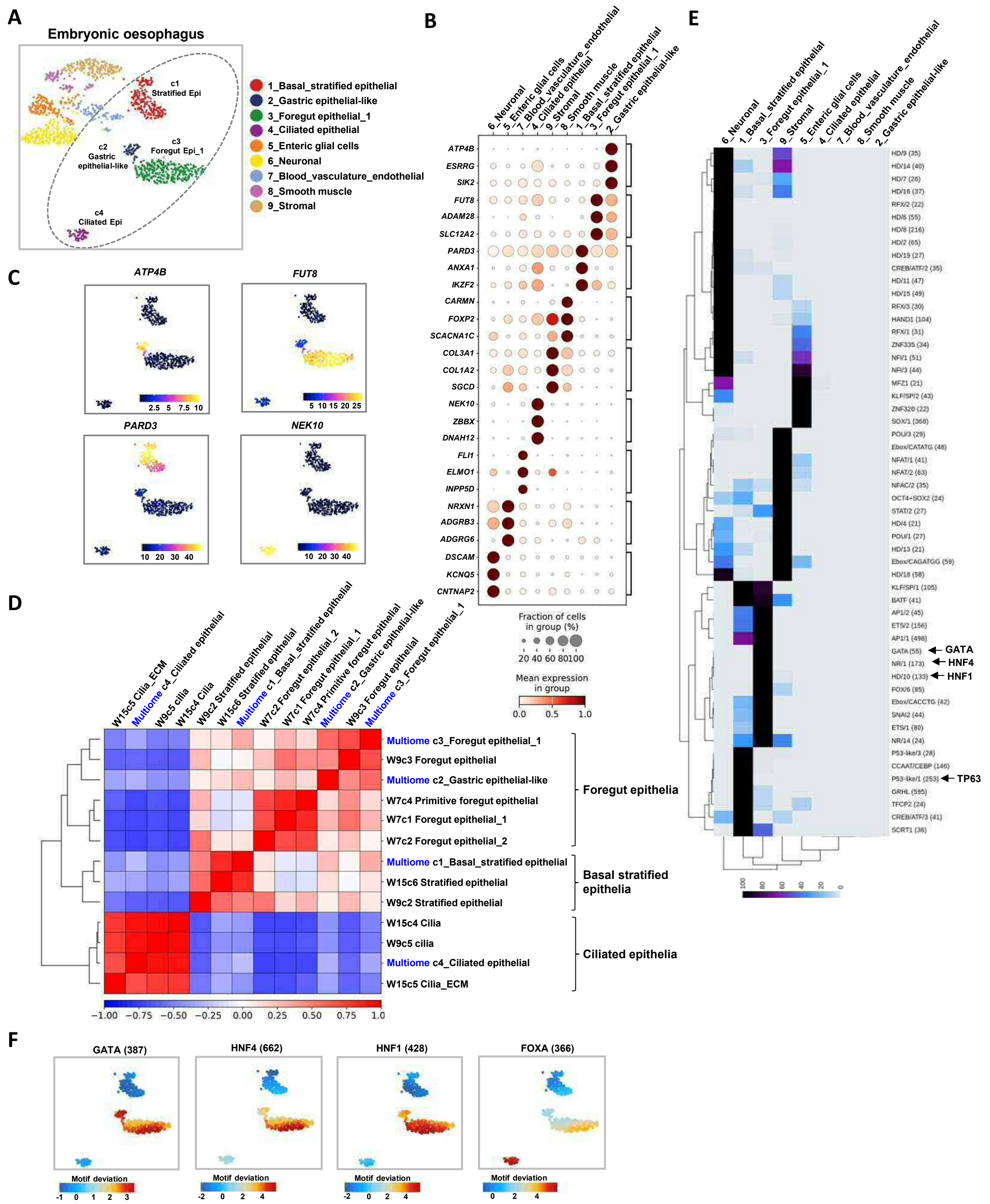

Fig. S11. Multiome analysis of the developing oesophagus at the embryonic-fetal transition. (A) UMAP of embryonic multiome cell clusters based on joint embedding of snATAC-seq and snRNA-seq. Major epithelial populations are annotated on the plot. (B) Dotplot of the relative average expression of three representative markers for each of the cell clusters found in the embryonic multiome data. The fraction of cells expressing each marker and relative expression levels (column normalised) are represented by the size and intensity respectively, of each dot. (C) Relative expression (indicated by scale bars) of the indicated genes projected on cells in the UMAP in part A. (D) Pearson's correlation plot comparing gene expression profiles of the epithelial cell clusters across developmental time points from Week 7, 9 and 15 snRNA-seq and embryonic multiome data. Broad categories of epithelial cell types are indicated. (E) Heatmap showing the relative enrichment of the indicated transcription factor binding motifs in each of the multiome cell clusters. Motifs discussed in the text are highlighted. (F) Transcription factor binding motif deviation scores for individual cells projected on the epithelial populations in the UMAP in part A.

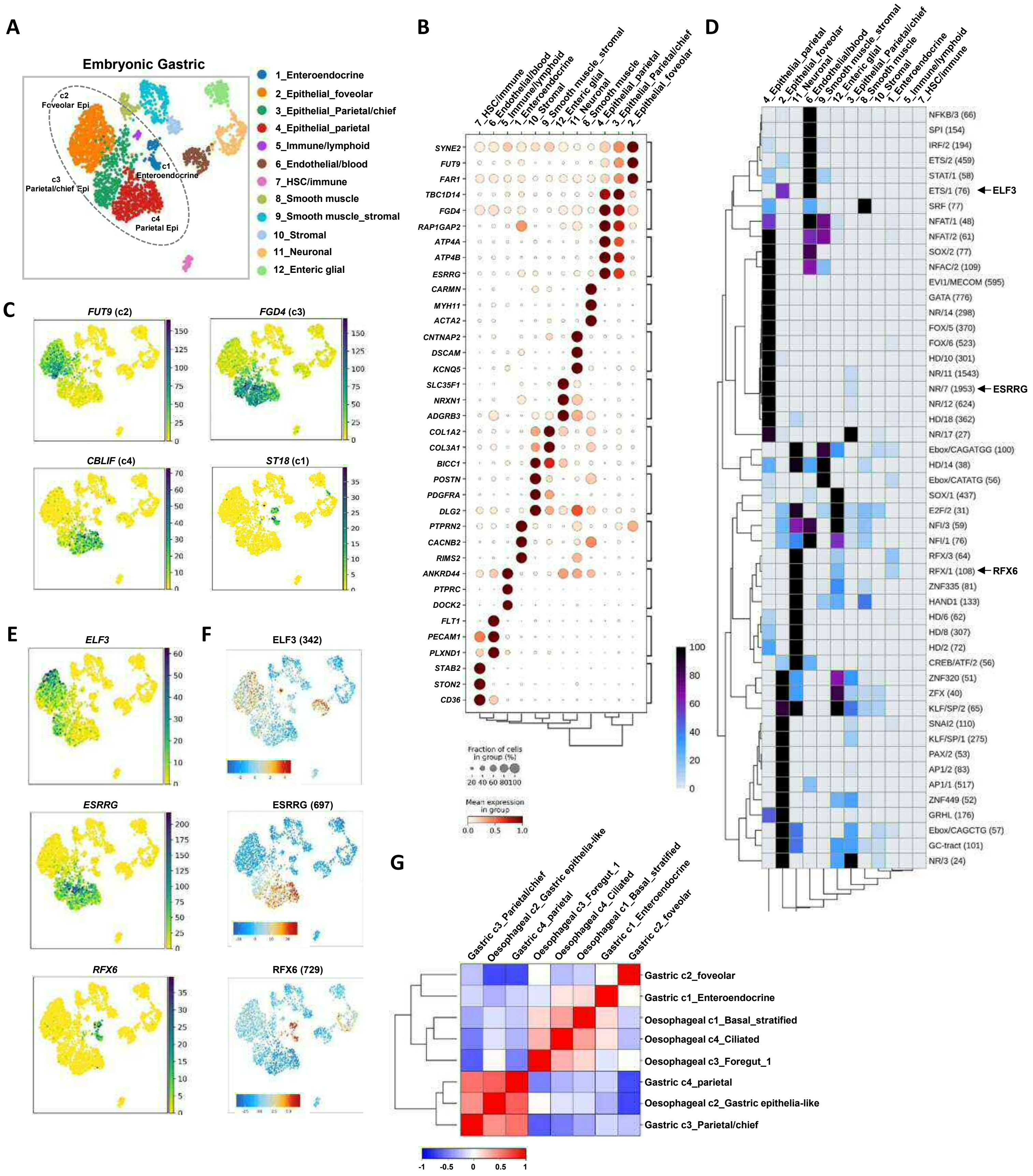

Fig. S12. Multiome analysis of the developing gastric cardia at the embryonic-fetal transition. (A) UMAP of embryonic multiome cell clusters based on joint embedding of snATAC-seq and snRNA-seq. Major epithelial populations are annotated on the plot. (B) Dotplot of the relative average expression of three representative markers for each of the cell clusters found in the embryonic multiome data. The fraction of cells expressing each marker and relative expression levels (column normalised) are represented by the size and intensity respectively, of each dot. (C) Relative expression (indicated by scale bars) of the indicated genes projected on cell clusters in the UMAP in part A. (D) Heatmap showing the relative enrichment of the indicated transcription factor binding motifs in each of the multiome cell clusters. Motifs discussed in the text are highlighted. (E) Relative expression (indicated by scale bars) of the indicated transcription factor encoding genes projected on cell clusters in the UMAP in part A. (F) Transcription factor binding motif deviation scores for individual cells projected on the epithelial populations in the UMAP in part A. (G) Pearson's correlation plot comparing gene expression profiles of the epithelial cell clusters from the embryonic oesophageal and gastric multiome data. Broad categories of epithelial cell types are indicated.

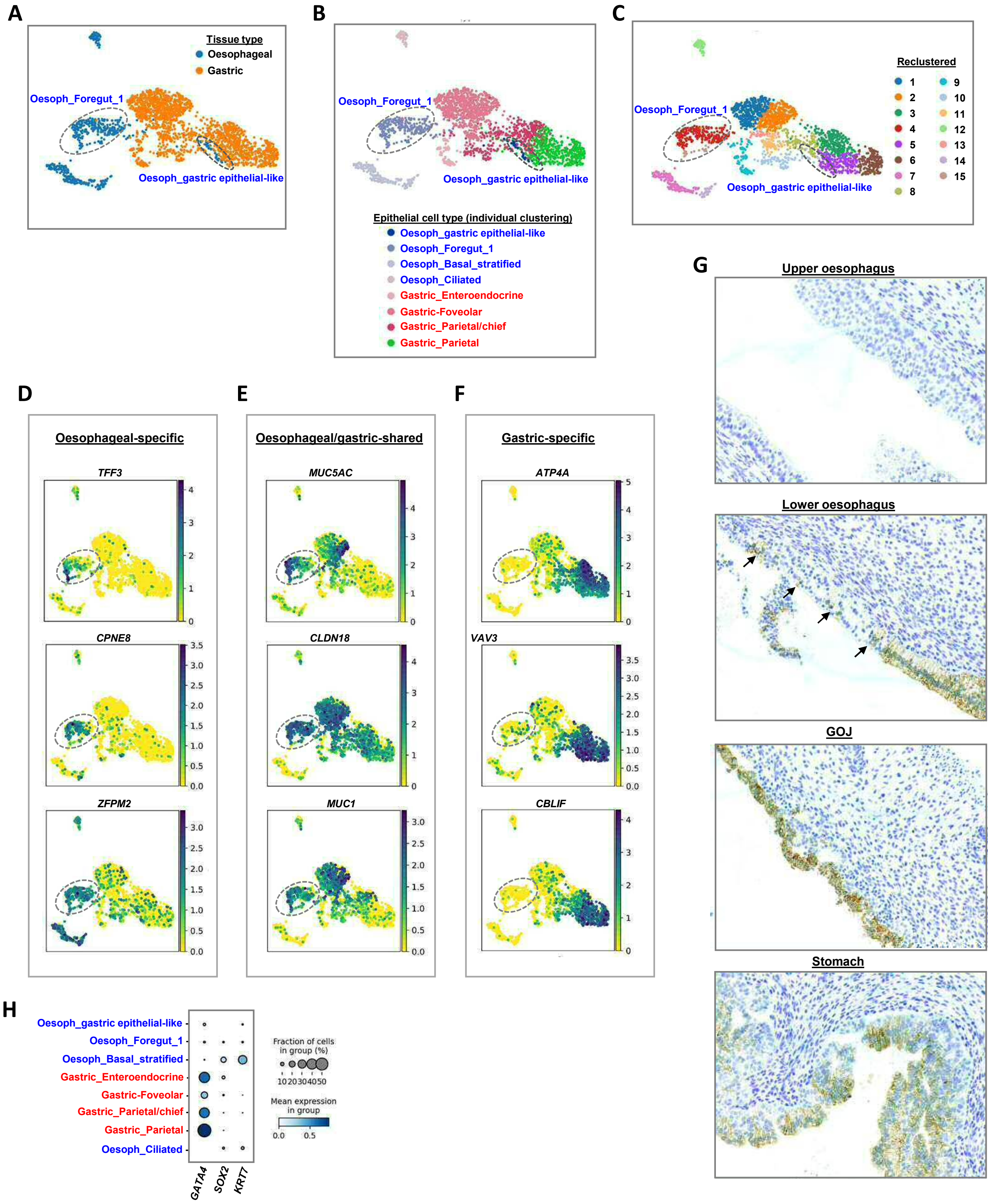

Fig. S13. Comparison of the developing gastric and oesophageal epithelial populations. (A-C) UMAPs of epithelial cells from both oesophageal and gastric multiomes clustered together based on joint modalities. UMAPs are coloured according to tissue type (A), original labelling of cell types from individual oesophageal or gastric multiomes (B) or new clusters generated from the combined datasets of epithelial cells (C). A population of cells from the oesophageal sample (oesophageal foregut_2) that closely associate with gastric cell-specific clusters is circled in blue on each UMAP. Oesophageal foregut_1 cells are circled in grey. (D-F) UMAPs of the epithelial cell clusters derived from joint clustering of oesophageal and gastric multiomes showing the expression of the indicated genes that characterise oesophageal foregut- specific (D), shared oesophageal and gastric (E) or gastric-specific genes (F). The oesophageal foregut_1 cell cluster is circled in grey. (G) IHC staining of MUC5AC expression in the indicated anatomical areas in a 9 week embryo x20 magnification). Arrows indicate patches of expression extending upwards from the lower oesophagus. (H) Dotplot showing the expression of the indicated genes in the epithelial cell clusters from the oesophagus (blue) or stomach (red).

Development • Supplementary information

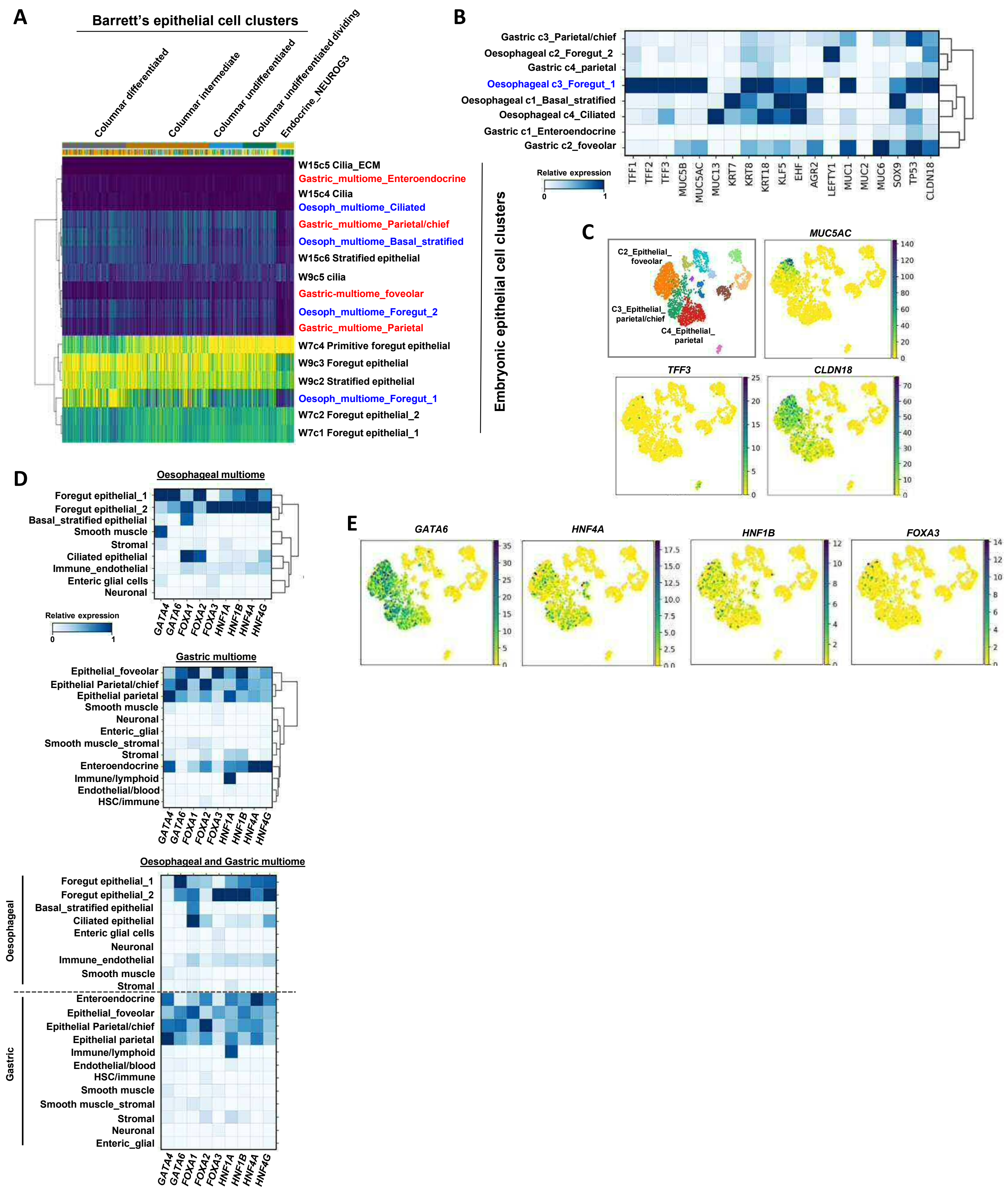

Fig. S14. Comparison of the developing gastric and oesophageal epithelial populations with Barrett's oesophagus. (A) Heatmap showing similarity scores between each cell in the epithelial cell clusters from adultBarrett's (x- axis) and the corresponding cell types found in different embryonic epithelial cell clusters (y-axis). (B and D) Heatmaps showing the relative expression (column normalised) of the indicated Barrett's marker genes (B) or the indicated transcription factors (D) in all epithelial clusters from the gastric and oesophageal multiomes (B) or each of the cell clusters from the embryonic oesophageal multiome data (D, top) the embryonic gastric multiome (D, middle) or both of the multiomes combined (D, bottom). (C and E) UMAPs of the epithelial cell clusters derived from gastric multiome (C, top left) showing the expression of the indicated Barrett's marker genes (C) or Barrett's core regulatory transcription factors (E). Major gastric epithelial cell clusters (c2-4) are labelled.

Development • Supplementary information

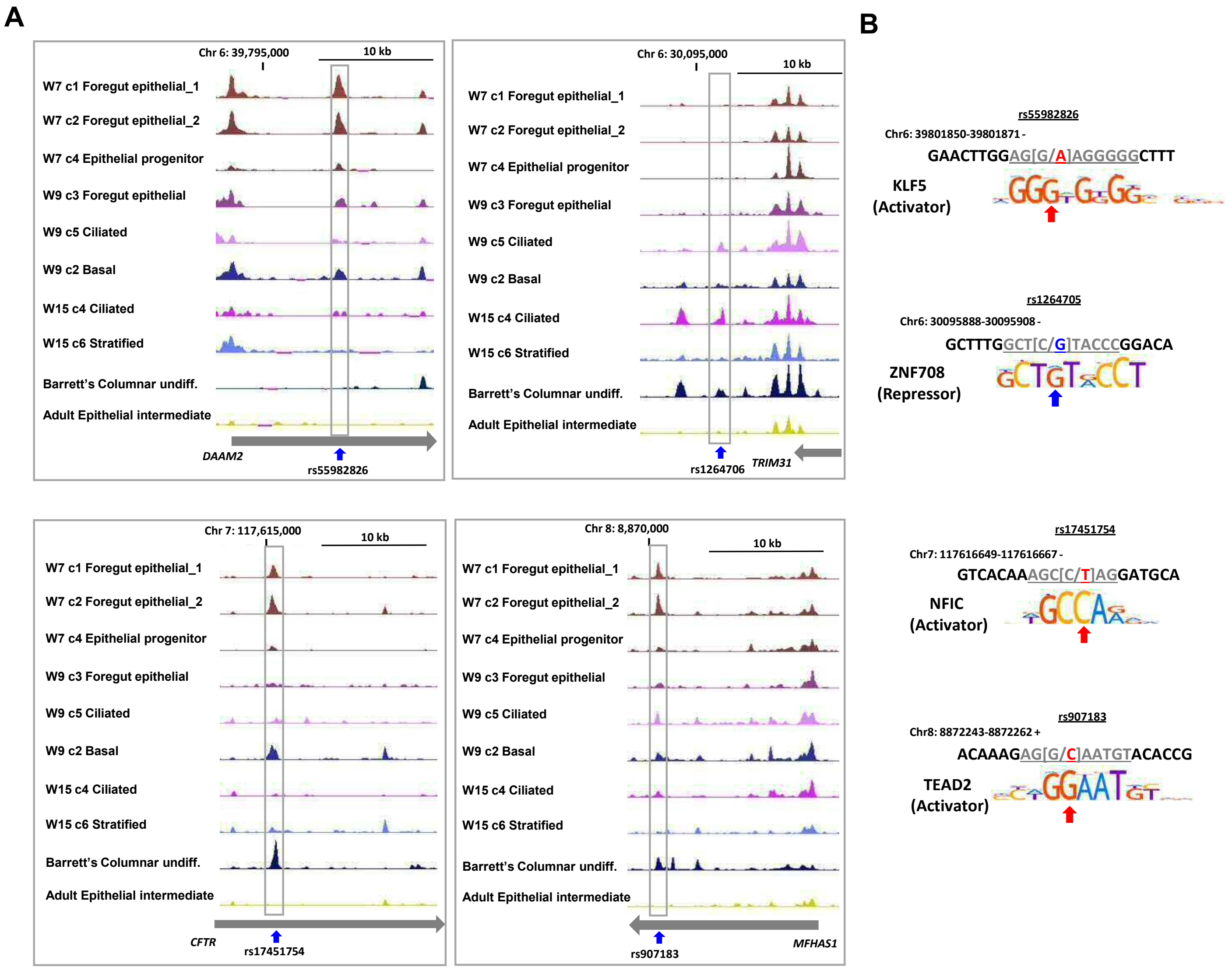

Fig. S15. Barrett's-associated SNPs map to enhancers in the developing oesophagus. (A) UCSC genome browser view of ATAC-seq signals surrounding SNPs rs55982826, rs1264706, rs17451754 and rs907183 in the indicated clusters of epithelial cells in human embryos, Barrett's undifferentiated columnar epithelia and adult oesophageal epithelial cells. The arrows below the tracks represent the directionality and extent of the genes covered by the views. Peaks containing the SNPs are boxed. (B) Sequences around significantly associated GWAS SNPs. The genomic location and DNA strand is shown above the sequences. Base changes are shown as WT/risk allele and the risk allele is coloured as red for disruption of an activator binding site or blue for creation of a repressor binding site. Logos for the DNA binding motifs of the indicated transcription factors are shown and corresponding bases in the DNA sequence are underlined. Arrows indicate the bases changed by the risk SNP.

Development • Supplementary information

Table S1. Marker gene lists for oesophageal cell populations at developmental stages (from scRNA-seq).

Available for download at
https://journals.biologists.com/dev/article-lookup/doi/10.1242/dev.204735#supplementary-data

Table S2. Marker gene lists for the developing oesophagus and stomach at the embryo-fetal transition (from multiome analysis).

Available for download at
https://journals.biologists.com/dev/article-lookup/doi/10.1242/dev.204735#supplementary-data

Table S3. Barrett's/OAC GWAS SNPs mapping to developmental enhancers.

Available for download at
https://journals.biologists.com/dev/article-lookup/doi/10.1242/dev.204735#supplementary-data

Table S4. Antibodies used in this study.

Antigen	Manufacturer	Ref Number	Dilution	Raised in
CLDN18	Abcam	ab203563	1:500	Rabbit
FOXJ1	Abcam	ab235445	1:1000	Rabbit
KRT4	Antibodies.com	A249123	1:500	Mouse
MUC5AC	Cell Signalling	61193T	1:400	Rabbit
MUC5AC	Novus	NBP2-15196	1:500-2000	Mouse
TFF3	Abcam	ab109104	1:500-2000	Rabbit
